# A feasibility study of Augmented Reality Intervention for Safety Education for farm parents and children

**DOI:** 10.3389/fpubh.2022.903933

**Published:** 2023-01-09

**Authors:** Kang Namkoong, John Leach, Junhan Chen, Jiawen Zhang, Bryan Weichelt

**Affiliations:** ^1^Department of Communication, University of Maryland, College Park, MD, United States; ^2^National Children's Center for Rural and Agricultural Health and Safety (NCCRAHS), Marshfield Clinic Research Institute, Marshfield, WI, United States

**Keywords:** augmented reality, AR, agricultural safety, farm safety, children, public health intervention, feasibility, acceptability

## Abstract

Agriculture is one of the most dangerous U.S. occupations with high rates of injuries and fatalities, and especially more dangerous for children, having more young worker deaths than any other industry. Thus, safety education is essential in promoting safe and healthy working habits in agriculture. Augmented reality (AR) technology has great potential to enhance the effectiveness of safety education due to its high levels of system-user interactivity and media enjoyment. This study aims to: (1) develop Augmented Reality Intervention for Safety Education (ARISE), an AR 3D simulator that presents farm accident situations with immersive media technology, (2) examine the feasibility of ARISE, and (3) evaluate the potential of ARISE as an effective agricultural safety education program for farm parents and children. To test the feasibility of ARISE, we conducted semi-structured in-depth interviews with ten parent-child dyads at an extension office located in Maryland. Participants were farmers who owned and operated a family farm(s) with their child or children ages 5–13. The interviews included asking participants questions about their perceptions of farm risks, sources of risk education, and protection methods. In the next step, participants used ARISE with researcher guidance. After using the application, participants were asked questions about their experience using ARISE and suggestions for improvement. The interviews were then transcribed and analyzed following the conventional content analysis method. Three main themes emerged—*demand* (e.g., perceived risk and need for education; lack of farm safety education from school), *acceptability* (e.g., attitude toward AR technology; perceived realism; perceived ease of use; perceived usefulness), and *implementation*. These findings help us understand how an immersive experience can play an impactful role in enhancing agricultural safety. The feasibility of ARISE sheds light on the potential of AR technology for an innovative safety education program.

## Introduction

Agriculture is one of the most dangerous occupations with high rates of incidents and fatalities in the western world (1) and in the United States (2, 3). Agriculture is also more dangerous for children than any other demographic, having more younger worker deaths than any other industry and a much higher proportion of youth worker deaths compared to adult workers (4). Further, agricultural workplaces often double as home as well, which places young, non-working children in dangerous worksites. Thus, safety education is an essential component in promoting safe and healthy agricultural environments, but current safety education for children largely depends on parents' guidance and/or first-hand experience.

Augmented Reality (AR) technology has great potential to enhance the effectiveness of safety education due to its high levels of system-user interactivity and media enjoyment. AR technology allows computer-generated or extracted real-world sensory information (e.g., sound, video, or graphics) to be overlaid on a physical environment directly or indirectly in real-time (5–7). AR prototypes have been applied within agricultural safety and health, and specifically in the application of emergency response safety (8, 9). Further, AR has shown effectiveness in improving safety and health in occupational safety [e.g., (10, 11)] and in safety education for children [e.g., (12)]. Despite evidence of effectiveness, it has seldom been used for children's agricultural safety education. Thus, this study aims to: (1) develop Augmented Reality Intervention for Safety Education (ARISE), (2) examine feasibility of ARISE, and (3) evaluate the potential of ARISE as an effective agricultural safety education program for farm parents and children.

## Materials and methods

### Augmented reality and safety education

Augmented reality (AR) refers to “a live direct or an indirect view of a physical, real-world environment whose elements are augmented by computer-generated input, such as sounds, graphics, or GPS data” [(13), p. 1351]. Different from virtual reality (VR), which provides users with fully immersed experiences through a purely-synthesized virtual environment, AR brings a virtual object into the real-world setting, which aims to enhance the real-world experience with synthetic information *via* visualizations and audio (14). Augmented reality is more easily accessed by mobile electronic devices (e.g., tablet PCs and smartphones) and its users can control their presence in the real world, which helps enhance both virtual and real-world experiences.

In recent years, AR technology has been applied to a variety of fields such as agriculture [e.g., (15)], healthcare [e.g., (16)], education [e.g., (17)], behavioral science [e.g., (18)], food science [e.g., (19)], safety interventions [e.g., (20)], and journalism [e.g., (21)]. Among them, safety education is one of the fields in which AR has been widely adopted. For example, AR interventions have been developed and applied to driving simulation [e.g., (22–24)]; evacuation research under disasters (25); safety training in the construction (20); and health and safety intervention among elderly people (26, 27), specifically, how AR reduces fall risk in the elderly and navigate memory loss issues.

Past research confirms the potential of AR interventions for effective and practical safety education tools. For instance, Schall et al. (23) developed an AR safe driving education intervention and evaluated its effectiveness in detecting hazardous objects on roadways and directing elderly drivers' attention. Using AR cues in the interactive driving stimulators, they found AR can improve elders' driving safety by increasing hazard detection without affecting their other driving tasks. This study shows AR technology can mitigate the crash risk for elderly drivers. Chandrasekera et al. (26) created an augmented space for older adults that could potentially help solve their difficulty in living independently, primarily due to memory loss and physical impairment. They developed a hybrid space with an AR object location and information system. They found older people used the system with ease and were open to the idea of using such augmented space to enhance their living environment.

AR safety interventions also improve crisis or emergency management ability (25) and safety training in the construction industry (20). Lovreglio and Kinateder (25) conducted a systematic literature review about how AR was used to improve building evacuation when disasters such as fires, earthquakes, and tsunamis occurred. The result showed AR evacuation tools were effective in evacuation training, as the intervention enabled the tracking of user position, orientation, and input. This study showed the use of AR filled some gaps in improving evacuation effectiveness as compared to VR and other methods. Hasanzadeh et al. (20) delved into construction workers' risk behaviors and investigated whether providing passive haptics in the mix-reality setting could help capture workers' risk-taking behaviors, identify at-risk workers, and propose injury-preventative cues. Findings indicated that the immersive environment was suitable for triggering workers' behavioral change; meanwhile, it could help evaluate workers' risk perception and risk-taking behaviors in a risk-free setting.

In addition to the diverse topics and areas, AR safety education interventions have been developed and studied for diverse populations, such as people with reduced vision (28), older adults with physical impairments (26), and construction workers (20). However, children, more specifically farm children, have seldom been target populations of immersive media technology interventions, although they are much familiar with this type of technology; thus, AR technology has the potential to be an effective intervention for enhancing risk awareness and safety education.

### Development of Augmented Reality Intervention for Safety Education (ARISE)

ARISE is an AR 3D simulator that presents farm accident situations with immersive media technology. To create ARISE, we collected and analyzed cases of child-involved agricultural accidents obtained from the *AgInjuryNews* database (https://www.aginjurynews.org). Originally launched in 2015 and later redesigned in 2018, the AgInjuryNews database provides a growing collection of U.S. and Canadian agricultural injury and fatality reports, primarily sourced from news media (29, 30). Using the database, we searched news reports published in 2018 about agricultural injuries involving kids under 18 years old and identified dominant patterns of child-involved farm accidents. An analysis of the cases showed that the most common causes of youth farm injury and fatality include: (1) run over by tractors or trucks and (2) falling off or falling into farm machines. In addition, considering that many farms raise livestock animals, we added one additional risk scenario that involves livestock. Therefore, there are three risky scenarios in ARISE: tractor run-over, falling off a truck, and horse kicks (see Appendix 1 in Supplementary material).

Once we selected children-involved farm injury cases, we developed ARISE using ARKit with Xcode 9. ARKit is Apple's augmented reality framework that can run on any iOS device. ARKit uses VIO (Visual Inertial Odometry) to accurately track the world around the device, which enables a realistic user experience. Xcode is IDE (Integrated Development Environment) for iOS, which provides software development tools to create augmented reality applications using ARKit framework.

### In-depth interviews and analysis procedures

To test the feasibility of ARISE, we conducted semi-structured in-depth interviews with ten parent-child dyads on November 9th and 16th, 2019 at an extension office located in Maryland. Eligible participants were farmers who owned and operated a family farm(s) with their children aged 5–13. Table 1 shows participants' nicknames and their demographic and farm information. The interview consisted of three parts. In the first part, we asked participants a series of questions about their perceptions of farm risks, sources of risk education, and protection methods. In the second part, we asked participants to use ARISE following our guidance. After participants used the application, we asked them questions about their experience of using ARISE (e.g., the usability of ARISE and its usefulness for safety education) and suggestions for improving ARISE (see Appendix 2 in Supplementary material for the interview protocol). The interview and testing process lasted approximately one hour. On average, the participants used ARISE for about 10 mins, and pre- and post-intervention interviews took about 20 and 30 mins, respectively. At completion, participants were thanked and offered a $100 gift card for their participation.

**Table 1:** Demographic and farm information of participants.

Participants' names^*^	**Farm type and size**	**Machines on the farm**
LeAnne and Dillion (8-year-old boy)	Split households with two farms: (1) a 14-acres farm with cats, chickens, goats, and lambs, and (2) a 1,000-acres farm with cow-calf operation, feeding out Angus operation, hay operation, and crops growing	Lawnmowers, tractors, skid loaders, telehandlers, and trucks
Rick and Hayden	460-acre farm with grain and chicken	Tractors, big trucks, tractor trailers, some construction equipment
Mary and Bailey (11-year-old girl)	100-acre farm for grain elevator	Tractors, trucks, skid loaders, and conveyer systems
Sherry and Paige (9-year-old girl)	10-acre farm with horses, goats, chickens, dogs, and a garden	Rangers, four wheelers, lawnmowers, tractors, wagons
Carey and Brian (5-year-old boy)	250-acre farm with pigs and grains	Combines, tractors, drop trailers, trucks, and tractor trailers
Lydia and Caleb (9-year-old boy)	Part of a big family farm with chickens, horses, pigs, and grains	Combines, tractors, spreaders, and sprayers
Kate and Colton (10-year-old boy)	A 58-acre farm (two sections) with cows, longhorns, pigs, goats, llamas, alpacas, and chickens	Hay balers, hay wagons, tractors, poultry equipment
Emilia and Waverly (12-year-old girl)	A dairy farm with 140 milk cows	Tractors, tractor trailers, trucks, feeders, mixer wagons, and skid steers
Caroline, Hallie (13-year-old girl), and Mackenzie (12-year-old girl)	A small farm growing vegetables and raise a few animals including pigs and lambs	Tractors and zero turn mowers
Chelsea and Kiley (9-year-old girl)	A hobby farm with horses, steers, goats, chickens, dogs, cats, and pigs	No machinery at the moment of the interview. They had a tractor and a bush hog before

* Nicknames have been used to protect participants' privacy.

The interviews were then transcribed and analyzed following the conventional content analysis method (31). We employed this method because this study aims to describe the status of agricultural safety education and the feasibility of using ARISE for agriculture safety education. First, three researchers independently open-coded three interview transcripts. In this step, we adopted a general inductive approach (32)—the coding categories were not predefined by the researchers but instead emerged from the data. Reading the transcripts in detail, the three coders paid attention to the text relevant to the research questions. From the general inductive approach, preliminary category labels were identified with a word or short phrases from the interview transcripts. The researchers also marked the examples that illustrated the meaning of the preliminary categories. After coding the three transcripts, the researchers met to discuss the categories and examples they found. The common categories were documented and used as a coding framework for the remaining transcripts. The researchers then coded the remaining interviews and re-coded the original three interviews using the coding framework. After that, the researchers met again to discuss the results. Categories with similar meanings were grouped into superordinate themes, which were used to organize the writing of the results.

## Results

Demand, acceptability, and implementation are three general areas of focus addressed by feasibility studies (33). The results section is organized to address the three areas. The interview showed that children mostly learned about farm safety from family members. There was a lack of school education and innovative interventions on farm safety, which led to the demand for farm safety education interventions. ARISE has the potential to fulfill the demand because respondents showed great acceptance of ARISE and intentions to implement ARISE within their families and communities. To protect the participants' privacy, we used nicknames when quoting their comments in the following sections.

### Demand of agricultural safety education for children living on a farm

Investigation of demand involves understanding the extent to which a new program is likely to be used (33). It involves understanding recipients' needs or actual use of particular interventions. In the interview, respondents showed high-risk perceptions and agreement on the importance of farm safety education. However, we found that farm safety education for children primarily relied on first-hand experience with family education, second-hand experience from local communities and media content, and 4H clubs. Respondents were disappointed about the lack of school-based agricultural safety education, which indicated the demand for farm safety education interventions.

#### Perceived risk and need for education

The interview showed that parents understood the risks that could happen to children and the importance of teaching children about farm safety. They mentioned that “there's always a risk” (Kate). They were “even losing sleep at night” because “a worst-case scenario goes through my mind” (Sherry). Respondents emphasized that “as a parent, you have to instill safety in your children” (Kate) and “fill their heads with knowledge” (Caroline). Caroline is very cognizant of the potential dangers of working in agriculture. She admits, “it's dangerous for anybody. I mean, lots of farm accidents are going to happen, so we just have to do our best, train them and just keep telling them over and over again… they have to have a little instruction.”

#### Family education and first-hand experience as primary sources

To combat fearful feelings of what could happen, parents and other close family members, such as grandparents and uncles, took responsibility and became the primary source of children's agricultural safety education. Families reported that they have used education strategies, including having open communication about potential risks, keeping leery of hazards, and setting rules. During the interview, it was evident that parents had had many conversations about risks with their kids. For example, LeAnne directed this comment to her grandson, Dillion, an 8-year-old boy, “we spent a lot of time talking about that stuff, and he knew then to stay away from it, the electric fence.” Another parent noted that they “constantly remind them [kids] this is dangerous” (Kate) and “always tell them the right thing, what to stay away from… tell them what can happen” (Carey). Another participant mentioned that family members have kept “leery of all the things going around him,” referring to the child (Mary), and kept kids “always under adult supervision” (Kate).

Kate specifically noted that she also constantly reminds her children how their environment can be dangerous. She gave us an example saying, “Just yesterday, we have an RTV, which is like a utility vehicle that we drive around… And my little one there, he likes to [play] like a wild cowboy and hang off the side of it sometimes. And I'm always like, you're going to fall, you're going to get hurt. And like as a parent you're almost like sweating.” Lastly, families set rules for children to keep them from risk. For example, children were told “don't run out of the yard, don't play under the trucks, even when they're parked, don't get underneath or around or like sit in a tire” (LeAnne). For another example, as Beth said, “if I'm in a tractor and they get off of the school bus, they know that they need to go in the house.”

In addition to getting knowledge from family members, children also learned about agricultural safety through “observations [of] and paying attention [to]” (Hallie, a 13-year-old girl) first-hand experience. During the interview, children and parents shared first-hand dangerous experiences they had and how they learned from them. The experiences ranged from unpredictable animal movements to grain dust explosions. A first-hand experience could be a close-call or near miss. For example, Emilia reported almost being involved in an incident the day before the interview. She said, “I'm a little more protective over it … A lot of the younger kids that are driving the tractor their parents have grown up on a farm, so they don't think of the silly things that I think of. Like, what if it starts sliding? And their little brain gets really nervous where … they end up in the ditch… I mean, I almost had it happen to me yesterday, so luckily, I could get it stopped, but I just think … if you aren't old enough, you don't have the brains to react as quickly.”

Similarly, Rick and Hayden reported a dangerous situation highlighting incidents are possible even for seasoned farmers. The father said, “I had a close call. Almost ran over him (Hayden) one time. I had come up into the yard with the tractor, and he was fooling around on the computer. And she (Rick' wife) said she was going to send him out, but I didn't know. He had come up there, and I didn't even see him. Luckily my wife had come out there with him, and she grabbed him, pulled him out of the way. I wouldn't have run him with the tractor, but the implement behind me probably would've hit him.”

Colton described an experience when animals chased him and his friends because they wanted to load the animal up. He realized the dangerousness of the experience and said, “I don't think I'll ever do that again.” A first-hand experience can also be incidents where an injury did occur. Lydia described this memory to Caleb, a 9-year-old boy, “you jumped off of something in the shed and hit a piece of equipment and cut your leg. Got stitches.” The interviews implied that due to their family farming culture, they learn about risks on a day-to-day basis and through the repetition of daily tasks.

#### Second-hand experience as supplemental sources

Second-hand experience from local communities and media content provided supplementary resources to enhance children's safety education. The second-hand experience was primarily from local communities and used to educate children on safety practices. Chelsea described how hearing about a lawnmower incident led her to tell her children, “Remember when we heard about the little young kid that got run over with a lawnmower? They got injured. I don't know if you might have been too young, but we talked about that. You have to be really careful.” She followed up this incident with another memory saying, “and then a friend of ours got hit with a rock from I think a bush hog or mower.” After detailing this incident, she looked to her child and said, “that's why we don't come here when the mower [is] going.” These second-hand experiences often were tragic. Lydia made a point to share these stories with her children, “Anytime I hear something, I tell them about it. It wasn't that long ago a girl was killed… on a dairy farm… she might've fallen into a grain tank or something.” She continued with the notion that “I want them to be scared.” She saw this as an effective method for her children to accept the severity of their environmental dangers. While not experienced directly, undoubtedly, community storytelling was used as an educational technique.

Media content also provided plenty of second-hand experiences to learn from. Videos from social media allowed participants to display the dangerous nature of their life. YouTube was a valid source for the visual representation of risk. Rick explained this tactic when he said, “I've actually shown them some stuff on YouTube.” He went into detail when describing how dangerous PTO shafts are and the caution required when being around them. The use of YouTube was also common for Caroline, who actively looked for agricultural safety content on social media. “We watch a lot of tractor videos,” Caroline said. These parents found that having their children see these hazards enhanced their sense of danger. The use of media was not confined to YouTube. When speaking of her husband, Beth said, “he's really big into Twitter and that kind of stuff. I'm not really, but he does, and he'll tell me stories.” They discussed news, but it “depends on how severe it is, if we would share it with the kids or not. Cause you don't want to have them nervous that their dad's doing the same thing.”

Sherry spoke about how the media report she saw was actually happening in her community. “They had a really good documentary a while back about the grain bin safety, and that's been something that a number of our local fire companies have participated in. They've done some outreach, even with kids; I know growing up, a loaded tractor-trailer would sit there overnight or in a day. I remember playing in it, you could get up top and play in it, but you could get injured.” Sherry and Paige, a 9-year-old girl, did follow this up with a moment of media and second-hand experience convergence. Sherry started by saying, “oftentimes, I'll show them things I see on Facebook… there's one really good graphic of somebody's foot and you can see the hoof, and they have stitches where the horse stepped on their foot.” Paige followed up by saying that her friend broke her foot because of a similar instance. Moments of convergence such as these potentially show how mediated experiences can complement real-life experiences in enhancing agricultural safety. After discussing the second-hand experience with family, they “keep that precaution in mind and learn from mistakes” (Hallie).

#### Lack of farm safety education from school

“Sad” is how Mary described the lack of agricultural education in her children's school system. When asked about the education provided in local school systems, Mary indicated that the only way that children can learn is from their parents. Mackenzie, a 12-year-old girl, stated, “I don't think in regular school we ever really learned about tractor safety. It's mostly from our parents.” Waverly, a 12-year-old girl, supported this when saying, “it's mostly through my parents or family. Like a few times in kindergarten, we watched like a video.” Hayden explained one unique experience where his class went on a field trip to a dairy farm, but he largely felt that his school ignored the topic of farm safety. Lydia even explicitly asked her child about farm safety education at his school. She asked, “have you ever learned anything in school?” Caleb responded simply with “no.” The parents defaulted to the notion that schools need to do more. Kate gave her critique stating, “they need to do that [include agricultural curriculum]. There should be something cause there [are] a lot of kids, especially in the area where we live. They all live on farms.”

The lack of schooling on agricultural safety was partly made up for by the education provided by organizations such as 4H. 4H clubs provided kids interested in agriculture with an avenue to interact and learn from others within the agriculture community. Caroline described 4H as the only organizational learning as it's “really hard to get agriculture into schools.” This lack of institution-based education requires parents and children to actively join specialized organizations if they want to receive more systematic instruction on agricultural safety.

However, the respondents reported a mixed reaction when discussing the educational value of 4H. On the one hand, 4H provided advantages over family-oriented education. Carey, who taught a tractor safety certification class to teens in a 4H club, described how the 4H platform was effective in presenting statistics of agricultural injuries that increased their risk perception. On the other hand, some respondents reported drawbacks of 4H education. Mary admitted the inability of 4H to adequately cover all areas of agricultural safety education. She stated, “they talk about the safety of animals …, little things like that. Not really equipment. They do have a driver's safety class.” While the inclusion of the driver's safety class is necessary, Mary downplayed the significance as the class is for “fifteen (years of age) and up” and she described how farm children are already operating equipment from a much younger age. The driver's safety course offered by 4H was seen more as a legal requirement by Mary as children can “drive [equipment] on their own farm.” LeAnne admitted similar experiences that although the educational materials provided by 4H were important, implementing farm safety education at 4H was very much secondary to family-based learning.

In summary, parent participants perceived farm safety education as their sole responsibility, and their demand for school-based safety education was rarely fulfilled. This increased farm parents' responsibility to protect their children and other children in their agricultural community.

### ARISE acceptability

Acceptability refers to how the intended recipients—both targeted recipients and those involved in implementation—react to the intervention (33). In this study, we aimed to learn how farm kids (i.e., targeted recipients) and their parents (i.e., people involved in implementing ARISE) react to ARISE application. Based on the interviews, we learned about their attitudes toward AR technology and their perceived realism, perceived ease of use, and perceived usefulness of ARISE application.

#### Attitude toward AR technology

##### Favorable attitude: Unique, fun, and attractive

Respondents generally showed a favorable attitude toward using AR technology for agriculture safety education. Respondents endorsed the use of AR technology because it is more fun and attractive than other formats of education materials. They thought it was “pretty cool” to see the virtual farm on the table (Mary); the handling was “very unique” compared to other non-AR applications (Sherry); it was “fun” to use ARISE for safety education (Kate). As Rick said, “I think that could probably be seen by a lot more people, because people do like to. If that thing was on, say, Facebook, and it had some link about, watch this for farm safety, people would click on it, vs. if they saw some doofus standing up there, trying to do some demonstration.”

The attractiveness advantage of AR technology is essential for education targeting kids. Younger kids “have such a short attention span at this age” (Carey), and “farm safety isn't a super exciting topic (for them). If kids had to pick out, what do you want to learn today, I doubt farm safety would be at the top of their list” (Sherry). But because “kids are sucked into electronics” (Mary), a safety education app using AR technology “would probably capture a younger audience... capture attention a lot more than a link to click on a real-life video” (Carey). Lydia also felt that the AR application could “grab the attention more” compared to an adult just telling them how to be safe. Sherry and her daughter, Paige, described an imagined scenario showing how kids would be excited about using AR technology for safety education:

Sherry said, “If the kids go to a 4-H program…I could see them being excited coming home and saying I got to use an iPad. So instead of I say, ‘Hey, what'd you do today at school?' And it's-”

Paige responded, “I wrote a whole essay.”

Sherry agreed and said, “Yeah, I wrote a paragraph about farm safety as opposed to-”

Paige said, “I learned about farm safety, using an iPad!”

##### A second thought: Real-world demonstration and anti-electronics

While hesitant that AR interventions would work for their generation, the adults see the potential for their children. Caroline voiced this by saying, “as a parent, I think I would buy this app.” However, respondents showed a few concerns about using AR technology, although they also admitted that it is the way of learning for their kids' generation. Rick feared that some audiences like him would prefer a non-immersive mode of communication, “I'm kind of a real-world type person. I actually like real demonstrations, more hands-on type stuff.” But when being asked if he thought a video recording of a real-world demonstration is better, he said, “I think so. That's just my opinion, but I also grew up learning like that. This generation, maybe not so. They learn a lot from video games and stuff on iPads.” Similarly, Mary had concerns about kids using electronics too much. “I'm anti-electronics,” she said, “So I really don't think my kids need to be on app any more than they have to be.” With a second thought, she also said, “But that's technology. That's the world today. You know, I'm just old school.”

#### Perceived realism

For ARISE to be successful, the farm and the accident scenarios need to be perceived as parallel to real-world farm and risk scenarios. Sherry found that the ARISE farm was realistic. She said, “when you think of a farm, that's probably something that comes to mind.” Paige echoed that the AR experience “looks really real.” Kate also thought the virtual farm was “neat” because “standing there, you're looking around, you seeing this farm, which a lot of children that do live on farms can relate to everything going on.”

The risk scenarios presented in ARISE were also regarded as realistic and very much present on many farms. Kate described them as “real life scenarios.” Lydia stated that they were “100%” realistic about what was on the farm. Sherry provided a more descriptive statement, “I think having a tractor or the animal and the vehicle are very accurate portrayals of what type of activity would be going on.”

#### Perceived ease of use

The respondents generally agreed that ARISE was easy to use. They described it as “user friendly” (LeAnne) and “easy to maneuver through, and is interactive to keep their attention” (Hallie). LeAnne said, “I think it's easy enough to hold and walk around…Look here. Tap there. Watch.” They also found great use of the ability to zoom in and enjoyed “explor[ing]” (Paige) the AR farm from many different angles and perspectives. As Bailey, an 11-year-old girl, said, what she liked about ARISE most was “how you can move around and see how [it look] like in zoom in, instead of just sitting.”

#### Perceived usefulness

The respondents thought ARISE could be useful for safety education because children could easily understand the risk scenarios presented in ARISE and connect what they watched during ARISE usage to their own experiences. Dillion, an 8-year-old boy, explained that he prepared to look for the wrongdoings when he started to use the application, “when I got it, when I saw all the parts of it, I knew there was going to be something wrong. I knew if it was going to be one, two, or three, but I knew at least one thing was going to be wrong.” Kate also stated that her 10-year-old son, Colton, “knew right away before even start[ing] your scenarios.” After using the application, the kids interviewed could describe what went wrong in the risk scenarios. Mackenzie said, “I think it's descriptive enough to get their attention and clear enough to get the points through.”

Respondents stated that younger kids and kids from the non-farm area would be the ideal target audience for the application. They thought that ARISE would be useful, “especially for elementary age” (Mackenzie) or “pre-K, kindergarten, first grade, second grade” (Lydia). This met the intended audience that ARISE was designed for.

Surprisingly, some respondents felt that ARISE was more beneficial for “the non-farm people rather than the farm community kids” (Mary) because “they [farm community kids] have started early and they understand it's the outside world that is clueless about anything.” LeAnne described it as a unique sense of safety awareness by saying, “Because it's so repetitive and it becomes second nature to him. … he pays more attention to it because he's seen it forever … his awareness of what's going on, even if he's not telling you, but deep in here somewhere he notices and remembers that stuff.” Hallie outlined, “we learned about some things like… how to stay safe. Even for the kids that aren't as active (in farming), they learned about it when they go into that situation. Just observations and paying attention.” Emilia supported the idea that growing up surrounded by the risks of agricultural work serves as a necessary preparation. She does not fear for her own family but senses risks with visitors. She said, “for my children, no [perceptions of risk], because we are very pro-farm safety. Visitors, we tend to kind of keep them a little bit. We try to give them a run-through of like, if you see this coming, move away from the lane, that kind of stuff, it's more outside than our children we're worried about.” She thought those new to a farm or visiting a farm could use ARISE to start the conversation on safety. In Carey's words, ARISE would best be used to “spread awareness” to those with little to no knowledge of agricultural life.

### ARISE implementation

Implementation is an essential aspect of feasibility and is defined as the extent to which a program can be successfully delivered to intended participants to achieve its goal (33). Inferences of implementation are made through two subthemes: the likelihood of implementing ARISE in schools and 4H clubs and the advantages of implementing ARISE compared to other educational materials.

Implementing ARISE into classrooms is feasible because the schools are fully equipped with technology and the kids are used to learning through technology. LeAnne mentioned that in the county where the interview was undertaken, “5th grade and up all have a Chromebook that they can take home with them. 3rd and 4th grades have a Chromebook assigned to them that stays at school. And all the kids, from kindergarten up, go through the computer education program.” Some respondents felt that implementation in schools that are situated in agricultural communities would benefit greatly. Kate displayed this when she said, “it could be a great program to go into the school. Especially in this area because there's a lot of farmers that have children or grandchildren, there's a lot around here.”

In addition to implementing ARISE in schools, the respondents also mentioned the potential to implement ARISE in 4H programs. Lydia felt that 4H would be the best place as children in this setting would all have a practical use for it as ARISE. She stated, “my thought would be 4H. I mean, there's a lot of the children, especially with the animals, a lot of them in our area are in 4H.” Respondents involved in organizing 4H events also talked about the potential to implement ARISE at 4H fairs. LeAnne said, “at our fair, our 4H club does an interactive learning tent. That's full of ag education, ag literacy kind of stuff. And we've talked about having a farm safety piece, but we've never been about to figure out a way to put it in the tent...” She also felt that children were the ideal target audience when she stated that “to me, it's obviously this type of program needs to be tailored toward children. Kids are around this sort of thing (AR technology).” She believes it would appeal to many children at 4H who come from an agricultural background.

Respondents also mentioned that using ARISE had advantages over other education materials. Compared to real-world learning, LeAnne stated that ARISE has implementation advantages, “especially in a situation where you can't take the farm into the classroom to teach them. So, it's definitely a cool thing. And all the schools have technology.” She also stated that with ARISE, “they (children) can see all around and do all kinds of stuff, and the movement is really helpful for a kid.” Carey talked about its portability, “You haul this stuff everywhere; with this, you just take the iPad in, and they can do it right there in the classroom, or afterschool, or daycares. I really like the portability of it, and the fact that you can update it and change it.” Similarly, when talking about other education materials about farm safety, LeAnne said, “I've looked at a bunch of them, and we've done some small stuff with the 4H club. The problem is, it's either too big of a setup. Where this is great because it's a tablet, or even if, put it on a phone app, where it's smaller technology, all the farm safety stuff you see is big. They want you to have big equipment so you could see it, which is great, but not practical.”

## Discussion

This study examines the feasibility of ARISE, an AR 3D simulator that presents farm accident situations (see Appendix 1 in Supplementary material), as an effective solution for farm children's agricultural safety education. ARISE presents three common causes of farm injuries and fatalities (i.e., a child at risk of getting run over by a combine/tractor, falling off a truck, and being kicked by a horse) on a virtual farm projected on a table. The risk scenarios in ARISE were chosen based on an analysis of agricultural injury news obtained from AginjuryNews.org database. Before and after using ARISE, ten dyads of a farm parent and their child share their daily lives on a farm and immersive experience with ARISE. Three themes—demand, acceptability, and implementation—emerged from the interviews, showing ARISE's feasibility and potential as an impactful agricultural safety intervention.

First, there was a high level of risk perception about farm safety issues and considerable demand for innovative agricultural safety education interventions for farm children. This demand was not fulfilled by schools or relevant institutions, which made farm parents or grandparents solely take responsibility for their children's safety education and training. Specifically, it was commonly reported that agricultural safety education mainly occurs through family norms and culture. The interview revealed farm parents were fully aware of the risks that could happen to children in an agricultural environment, and they taught their children how to handle risky situations. The farm parents agreed on the need and importance of farm safety education outside of the family. In addition, the family members largely depended on their first-hand experience and second-hand experience from local communities and media reports, not from institution-based agricultural safety education. The respondents expressed their disappointment about agricultural education being ignored in public school curricula, although the schools are located in agricultural communities. These demands imply that ARISE would be well accepted by the target populations, farm parents and children, if it provides supplemental resources for family-based and first-hand experience-based farm safety education.

Second, the respondents found ARISE to be very realistic, useful, and easy to use, and showed a favorable attitude toward ARISE. All these evaluations indicate a high level of the acceptability of ARISE, a key component of the feasibility of an intervention. Participants found ARISE to be very realistic—both immersive media content (i.e., a virtual farm and farmers/their children's appearances and actions) and selected incident scenarios. An encouraging result was farm children's ability to relate what they watched from ARISE with their own experiences. They recognized risky behaviors shown in ARISE and connected them to their daily lives on a farm.

Respondents also appreciated the user-friendly nature of the tool and endorsed ARISE as well-designed to maximize natural user-system interactions. Farm parents and their children showed a favorable attitude toward using AR technology for agriculture safety education, describing ARISE as fun to use and more attractive than other formats of farm education materials. This entertainment-like design was developed for young kids who usually have a short attention time. The respondents also highlighted that ARISE would be useful and practical for non-farm children as well, because they have very limited experience and knowledge about the danger of agricultural lives. According to the technology acceptance model (TAM), perceived ease of use, perceived usefulness, and attitude toward usage are key components that predict the acceptance of a new system (34), and users' level of acceptance is a crucial component of the feasibility of an intervention.

Finally, the in-depth interviews revealed a great potential of ARISE that can be successfully delivered to intended users in schools and 4H clubs to supplement the lack of institution-based agricultural safety education. This indicates the high likelihood of ARISE implementation, an essential aspect of feasibility, compared to other educational materials. According to the interviewees, implementing ARISE into classrooms and 4H programs is not only feasible but also desirable, because laptops and tablet PCs have been widely adopted in the public school systems. In addition, throughout the COVID-19 pandemic, children have been familiarized with different learning technologies and platforms.

The portability of ARISE was also indicated as a great advantage. 4H programs and community events were frequently recommended for an educational setting. For example, 4H fairs provide several interactive agricultural learning opportunities but seldom present effective agricultural safety programs, because it usually involves sizable equipment that is hard to put in an event tent. Given that ARISE is a mobile application operated *via* an iPad or a smartphone, use at these events seems feasible.

The interviews also raised some concerns. Some farm parents expressed apprehensions about using immersive media technology because they believed their children spent too much time with media technology, commonly referred to as screen time. They preferred to limit their children's media time, even if the purpose of the media use is educational. At the same time, however, they appreciated the purpose of using AR technology in this project and acknowledged it's somewhat inevitable for their children to use new media more in their generation. When we apply AR technology to children's education, we should consider how we could reduce parents' concerns about using immersive media technology.

It is also noteworthy that some parents regard their own farm environment as more dangerous for visitors than their children, thus, felt that ARISE was more beneficial for “the non-farm people rather than the farm community kids.” They showed confidence in their children's farm safety knowledge and practice because their children grew up on farms surrounded by the risks of agricultural work and they taught necessary preparation for handling those risks. Therefore, they argued that ARISE would be effective for those who are new to a farm or visiting a farm, and provide a good starting point for farm safety conversation. However, they believed ARISE would have a limited impact on their farm children. It might be true, but it might be a result of optimistic bias. It is evident that farm children are exposed to more agricultural hazards than non-farm children. Therefore, future research needs to examine if there would be different effects of ARISE between farm and non-farm children.

Although this study primarily used in-depth interviews and its' goal was not a generalization of the findings, it is evident that ten dyads of farm parent and child cannot represent the target populations of ARISE. To bolster and generalize our findings, future research needs to recruit more participants from the target populations and test the feasibility of ARISE with different research methods, such as a longitudinal panel survey or an experiment in a laboratory setting. Beyond ARISE, future study needs to expand the scope of AR applications to resolve other medical and safety concerns targeting a broader research population.

## Conclusion

This study sheds light on the potential of the AR technology for an innovative safety education program. ARISE can contribute to the prevention of agriculture-related injuries and fatalities by providing farm parents and children with a user-friendly platform and vivid second-hand experience of incidents that frequently occur on farms. This finding is more meaningful for agricultural safety education because there has been very limited AR intervention research targeting farm children.

## Data availability statement

The original contributions presented in the study are included in the article/Supplementary material, further inquiries can be directed to the corresponding author.

## Ethics statement

The studies involving human participants were reviewed and approved by Institutional Review Board (IRB), Division of Research, University of Maryland, College Park. Written informed consent to participate in this study was provided by the participants' legal guardian/ next of kin.

## Author contributions

KN initiated this project, led the research and development process about the Augmented Reality Intervention for Saftey Education (ARISE), and led the writing of this research manuscript. JL and JC assisted with all research procedures, especially intervention design and data collection, wrote this manuscript together, and especially the research methods and result parts. JZ wrote the literature view part of this manuscript. BW secured the grant with KN, as developing initial ideas and design of this intervention and wrote this manuscript together. All authors contributed to the article and approved the submitted version.
