# Supplementary material for: A feasibility study of Augmented Reality Intervention for Safety Education for farm parents and children
Source: Front Public Health. 2023 Jan 9;10:903933. doi: 10.3389/fpubh.2022.903933 (PMC9875325; doi:10.3389/fpubh.2022.903933)
Supplement: Supplementary file 1 [file Data_Sheet_1.docx]

**Appendix 1. Screenshots of ARISE**

| **ARISE Overview** | | |
| --- | --- | --- |
| **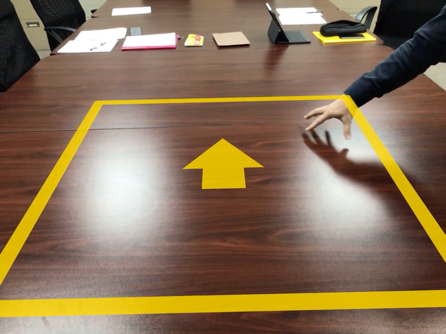** | **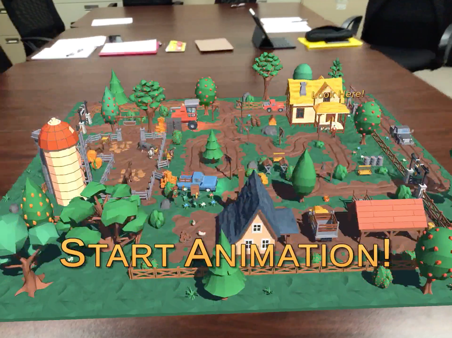** | **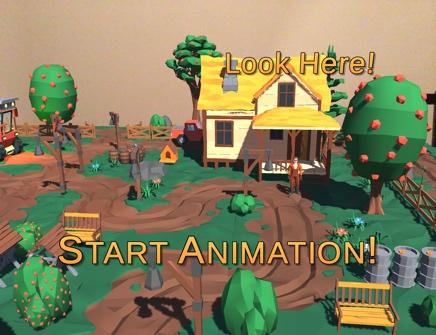** |
| **Scenario 1 (Tractor/Combine)** | | |
| **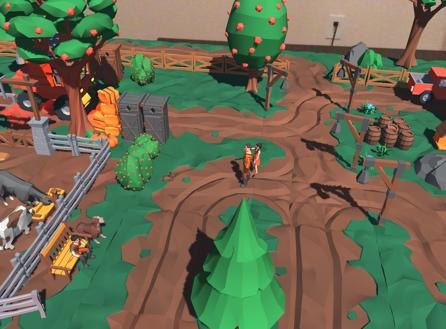** | **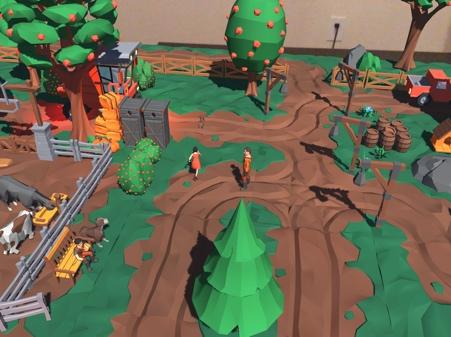** | **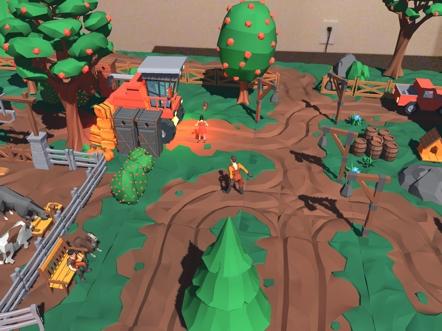** |
| **Scenario 2 (Animal)** | | |
| **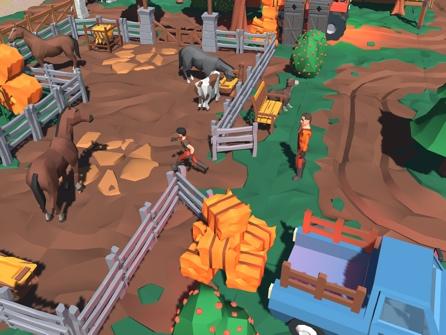** | **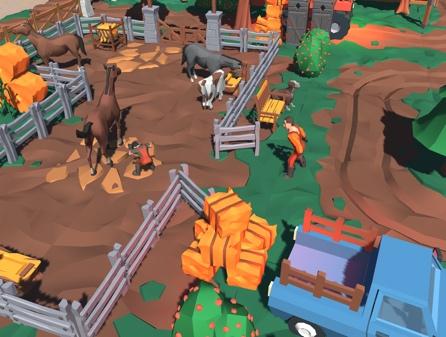** |  |
| **Scenario 3 (Pickup Truck)** | | |
| **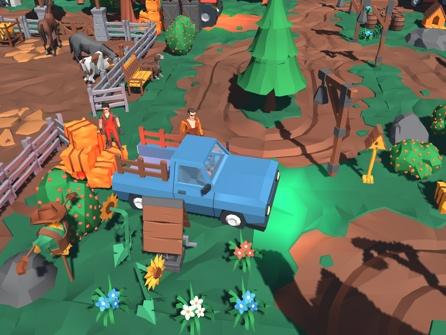** | **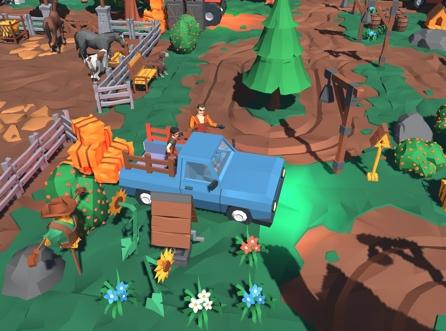** | **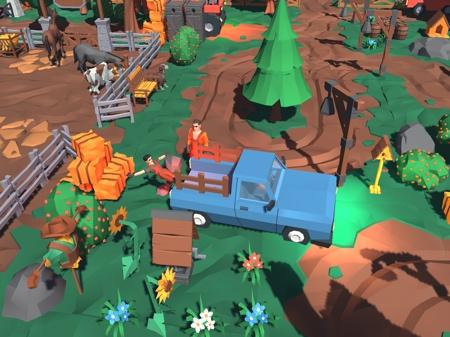** |

**Appendix 2. Interview Protocol**

Thank you for your time to participate in this study. The interview will last 40-60 minutes. During the interview, you will have a chance to use an AR Farm Safety Education Program (ARISE) for 5-10 minutes. Any potential loss of confidentiality will be minimized by storing electronic data in a password protected computer and hardcopy data in a locked cabinet of a locked office. I would like to ask your permission to record this interview for accuracy. Your participation is voluntary and you can decline to answer specific questions or to end your participation at any time.

[Consent form for face-to-face Interview]

Do you agree to participate and be audiotaped? [If yes, let the participant read and sign the consent form and continue. If the participant agrees to participate but not to be audiotaped, I will take notes instead. If the participant does not agree to participate, stop.]

**Questions**

**I. Background Information** (10-15 minutes)

1. Could you tell us something about the farm you owned? (Based on the answers, the follow-up questions could be: Do you have agricultural machines on the farm? How do you manage them? What does a typical day look like on your farm?)
2. Are there any potential risks to children on the farm? What are those risks? (Follow-up questions: Have you thought about a chance/likelihood your child could involve in a certain farm accident? Have you ever thought about the consequences/severity caused by those risks? How do you protect your children from those risks?)
3. Have you had any accidents or dangerous situations with your child in your farm? Have you heard of any accidents or dangerous situations for children in a farm in your community? (Second-hand)? Or from Media reports (e.g., Newspapers)?

**II. Intervention** (5-10 minutes)

1. Participants will use ARISE (Parent & Child & Parent/Child Together as talking to each other) before the following questions are asked.

**III. Questions about ARISE** (30-40 minutes)

1. Do you think ARISE can help in preventing your children from farm risks? If yes/no, why?
2. Could you compare ARISE to other forms of safety education materials (e.g., booklets, video clips, etc.) you have ever used/exposed?
3. Do you think using ARISE is a better safety education program? If yes/no, Why?
4. (For children) Do you like ARISE? Why you like/dislike them?
5. Is it easy or difficult to use ARISE?
   - Are there any difficulties related to operating/navigating ARISE?
   - Are there any difficulties related to understanding the scenario or the content about risks illustrated by ARISE?
   - Any suggestions to make ARISE easy to use?
     1. **Closing Questions**
6. Are there any other design features or content that you want to add to ARISE? Is there anything that you would like to add that I have not yet asked you about?
7. Demographic Information: Age / Gender / Race / # of children (age, gender), etc.?

**Note: Additional follow-up questions may be asked, as appropriate, with each participant.*

Thank you for your participation*.*
